# Association of Total and High Molecular Weight Adiponectin with Components of Metabolic Syndrome in Mexican Children

**DOI:** 10.4274/jcrpe.galenos.2019.2019.0113

**Published:** 2020-06-03

**Authors:** Javier A. Magaña Gomez, Daniela Moreno-Mascareño, Carla E. Angulo Rojo, Gisela Duarte de la Peña

**Affiliations:** 1 Universidad Autónoma de Sinaloa, Escuela de Nutrición y Gastronomía, Sinaloa, México; 2 Universidad Autónoma de Sinaloa, Facultad de Medicina, Centro de Investigación Aplicada a la Salud Pública, Laboratorio de Neurociencias, Sinaloa, México; 3 Universidad Autónoma de Occidente, Programa de Nutrición, Sinaloa, México

**Keywords:** Adiponectin, children, insulin resistance, metabolic syndrome, obesity

## Abstract

**Objective:**

Childhood obesity linked to metabolic alterations, tend to appear simultaneously with altered adipocytokines, suggesting a role in pathogenetic development. Low circulating level of total and high molecular weight (HMW) adiponectin have been associated with components of the metabolic syndrome (MetS) and could represent an independent risk factor with potential use as a biomarker. To examine the prevalence of MetS in Mexican school children and to investigate the association of total and HMW adiponectin levels with biochemical parameters related to MetS.

**Methods:**

The study included a population of boys and girls, from 8 to 11 years old. Anthropometric and biochemical parameters were evaluated according to weight and MetS status. A correlation analysis was fitted to establish an association between adiponectin concentrations and metabolic indicators.

**Results:**

One-hundred and fifty five children participated (59.4% females) from 8-11 years of age. The prevalence of MetS was of 10.3%. Impaired biochemical parameters, including total and HMW adiponectin, were associated with obesity. The adiponectin level was significantly lower in MetS than in non-MetS subjects (4.5 vs. 5.4 μg/mL). Total- but not HMW adiponectin concentration was negatively correlated with blood pressure, fasting insulin, fasting blood sugar and Homeostatic Model Assessment for Insulin Resistance.

**Conclusion:**

In young children, the total adiponectin level is associated with impaired biochemical parameters of carbohydrate metabolism and could be an excellent early predictor of metabolic complications.

**What is already known on this topic?**

Childhood obesity is related to several impaired biochemical parameters, including the concentration of total- and high molecular weight-adiponectin. A low adiponectin concentration was strongly associated with the prevalence of metabolic syndrome.

**What this study adds?**

The strong inverse correlation between adiponectin levels and biochemical parameters related to carbohydrate metabolism, contribute to the hypothesis that low adiponectin levels are associated with an elevated risk of diabetes. This reinforces the early role of insulin resistance in future vascular events. The circulating concentration of total adiponectin may represent an excellent biomarker to evaluate the risk of metabolic complications in young children.

## Introduction

Childhood obesity is a complex disorder, linked to metabolic and clinical abnormalities, such as insulin resistance, dyslipidemia, and hypertension. Various combinations of these impaired metabolic functions, even in children, have been used to define the metabolic syndrome (MetS) (1). The simultaneous occurrence of obesity and impaired metabolic functions demonstrates that the accumulation of adipose tissue is a frequent etiologic basis. Adipose tissue secretes numerous physiologically active peptides with properties similar to cytokines, commonly known as adipocytokines, such as leptin, interleukin-6, resistin, and adiponectin. While most of the adipocytokines promote dysregulated metabolism, adiponectin contributes to maintaining energy balance, insulin sensitivity, blood pressure, immunological processes, angiogenesis, fat metabolism, and homeostasis. When adiponectin levels are low, as occurs in central obesity, the risk for metabolic alterations increases in adults, adolescents, and children (2,3,4).

Circulating adiponectin exists as multimers of high-, medium-, and low-molecular-weight (HMW, MMW, and LMW, respectively), with predominantly HMW and LMW isoforms. In adults, low HMW adiponectin concentration reflects metabolic abnormalities related to obesity, insulin resistance, and vascular alterations more specifically than total-adiponectin (5).

Multifactorial disorders, such as MetS, may be affected by characteristics of the study population. In Japanese children, HMW adiponectin was inversely correlated with obesity and insulin resistance (6). Although the Mexican population is a heterogeneous genetic mix, significant heritability for adiponectin and obesity traits substantiate a genetic contribution to circulating cytokine levels in Hispanic children (7,8). Furthermore, the age profile of the population is an important factor related to the pathophysiology of MetS and adiponectin concentration (9). Therefore, this study was designed to investigate the association of total and HMW adiponectin levels with components of the MetS, and its possible role as an early risk marker in young Mexican children.

## Methods

### Subjects

Children between the ages of 8 and 11 were randomly selected to participate in a cross-sectional study from six representative elementary schools in five districts in a northwestern urban region of Mexico. Schools were selected from lists made available by the Educational Authorities, according to its geographical location. The protocol was presented to the school board, classrooms were selected, and parents were required to sign a written consent form to allow their children to participate. Children without medical therapy, with parental permission and who had fasted, were eligible for the study. The study protocol was approved by the Research Ethics Committee of the Faculty of Medicine, Autonomous University of Sinaloa, with registration number CONBIOÉTICA-25-CEI-003-20181012. All study procedures were in accordance with the 1964 Helsinki Declaration and its later amendments or comparable ethical standards. Volunteers were informed about the aim of the study, and written consent was obtained from their legal guardians.

### Anthropometric Variables

Anthropometric variables were measured according to standardized procedures (10). Body weight (BW) was measured with children wearing lightweight clothing and no shoes, to the nearest 0.1 kg using a standardized electronic digital scale (Tanita BC-553; Illinois, USA). Height was measured to the nearest 0.1 cm using a portable stadiometer (Seca-214; Hamburg, Germany) with the head in the Frankfort horizontal plane. Waist circumference (WC) was measured with a non-elastic, flexible measuring tape at the mid-point between the iliac crest and the lower edge of the ribs at the end of a normal expiration. Body mass index (BMI; kg/height in m^2^) was calculated and classified according to the age- and gender-specific cut-off points proposed by the World Health Organization (WHO) (11).

### Clinical and Metabolic Parameters

Systolic and diastolic blood pressures were obtained from the right arm with the child seated, after rest, using a digital sphygmomanometer and appropriately sized cuff. Venous blood samples were collected in the morning (8:00 to 9:00 am) by direct venipuncture after an overnight (10 to 12 hour) fast. Plasma and serum were separated by centrifugation, aliquoted, and immediately frozen at -80 ºC for later analysis. Glucose oxidase method (RANDOX Laboratories Ltd., Antrim, UK) was used to quantify fasting blood glucose levels. Triglyceride (TG), total cholesterol (TChol), high-density lipoprotein cholesterol (HDL-C) and low-density lipoprotein cholesterol (LDL-C) were measured using an enzymatic colorimetric method (RANDOX Laboratories Ltd., Antrim, UK). Insulin, total- and HMW-adiponectin, were measured by enzyme-linked immunosorbent assay using commercially available kits (ALPCO Immunoassays; NH, USA). Assays were conducted according to recommendations of the fabricant.

### Classification of Pediatric MetS

Currently, a standardized definition for MetS exists for adults, but not for children and adolescents. Therefore, modified WHO criteria were applied to diagnose MetS in children (12). This pediatric/adolescent definition requires either insulin resistance, hyperglycemia, or known diabetes plus the presence of two out of three other risk parameters: hypertension (elevated age/gender systolic and/or diastolic blood pressure ≥90^th^ percentile), dyslipidemia (hypertriglyceridemia ≥150 mg/dL or low-serum HDL-C <39 or <35 mg/dL in boys and girls, respectively), and central obesity (age/gender WC ≥90^th^ percentile or BMI ≥95^th^ percentile). The cut-offs for impaired fasting glucose were either ≥100 mg/dL or fasting insulin ≥75th percentile (13). Insulin resistance was defined using the homeostasis model assessment for insulin resistance (HOMA-IR), calculated as the product of the fasting plasma insulin level (µUI/mL) and the fasting plasma glucose level (mmol/L), divided by 22.5 (14).

### Statistical Analysis

 The distribution of data was assessed using the normality test Kolmogorov-Smirnov with Lilliefors correction. Data are presented as the means±standard deviation. T-test was used for comparison of continuous variables where applicable and by ANOVA with Tukey-Kramer *post hoc* comparison being used to evaluate group differences. For the variables without normal distribution, Kruskal-Wallis test for independent samples, according to BMI classification, was performed. Boys and girls were combined in the same groups because there were no significant sex-related differences in the anthropometric and biochemical data in the obese and non-obese children. Total- and HMW-adiponectin were correlated to anthropometric, biochemical, and clinical parameters using the Pearson or Spearman correlation coefficient. The statistical differences were considered significant at p<0.05. All statistical analyses were performed using the statistical software NCCS v.2007 (15).

## Results

**Figure 1:**
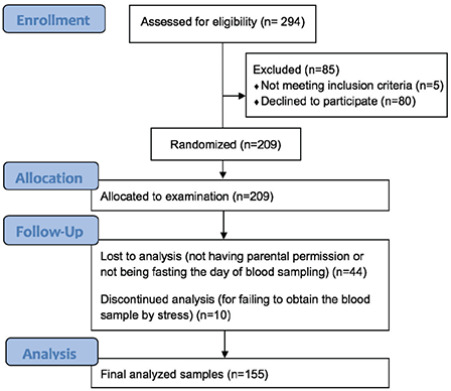
Flow chart of the recruitment stage of the study

The initial population consisted of 294 children, of whom 85 were excluded for not meeting the inclusion criteria or declined to participate, thus the drop out rate was 28.9%. On the day of blood sampling, 44 children were eliminated for not having parental permission or not being fasted, another ten were dismissed for failing to obtain the blood sample because of stress at the moment of sampling. There were no cases of children on medication or kidney disease excluded from the study. This resulted in a study cohort of 155 children (59.4% of females): 75 of healthy weight, 37 overweight, and 43 obese (Figure 1). The prevalence of MetS was 10.3%, according to the modified WHO definition. At the initial analysis, children showed no significant sex-related differences in the anthropometric and biochemical data; therefore, they were combined in the same groups. Characteristics of subjects and comparisons of mean values of clinical and metabolic continuous variables were analyzed according to obesity status (Table 1). Age was similar (p>0.05) between groups. In the obesity group, insulin and adiponectin had statistically higher concentrations (p<0.05), while HDL-C, total- and HMW-adiponectin were lowest. Also, blood pressure was higher in the obesity group.

When comparisons were made according to the presence or absence of MetS (Table 2), there was no difference for age (p>0.05). However, weight, BMI, and WC were different between groups (p<0.0001) with the highest values in the group with MetS. Insulin, HOMA-IR, LDL-C, TG, and blood pressure were significantly higher in the MetS group, while total adiponectin and HDL were significantly lower. Fasting blood glucose concentration, TChol, and HMW-adiponectin did not differ between the two groups.

Absolute values of adiponectin were tested by correlation analysis, and total-adiponectin had a significant negative correlation with anthropometric parameters and biochemical variables related to carbohydrate metabolism but not with those of the lipid metabolism (Table 3). In addition, a significant inverse correlation was found between total-adiponectin and the number of MetS components present. HMW-adiponectin was inversely correlated with weight, BMI, and HDL-C although no other significant correlation was found for the other parameters examined (Table 3).

## Discussion

The increase in childhood obesity worldwide is recognized as one of the most severe public health problems. The evidence of the association between childhood obesity and parameters of MetS is increasing (7,16,17). Adipocytokines and genetic background are known to be important in the pathogenesis of MetS (18,19). In the present study, we assessed the impact of childhood obesity and MetS in young Mexican children and its association with total- and HMW adiponectin.

Using the WHO definition, the prevalence of MetS found in this study (10.3%) was higher for the general child population compared with that found in other populations (3% to 8.4%) (20,21,22). However, it is difficult to contrast the prevalence of MetS because modified and non-standard definitions have been used and no globally accepted set of criteria exist for defining MetS in the pediatric/adolescent population. The prevalence of MetS in children and adolescents, based on the National Cholesterol Education Program’s Adult Treatment Panel 3 definition, tends to give higher prevalences and has been reported to vary from 4.2% to 18.6%, and in a similarly aged population (7-9 years old) to our study it was 15.8% (23,24,25,26). This increased prevalence highlights the importance of early diagnose of MetS in childhood, to prevent the progression from obesity to insulin resistance, cardiovascular disease, and type 2 diabetes.

The analysis of anthropometric variables according to weight status has confirmed that each component of the MetS worsens with increasing weight, independent of sex (27). As has been reported previously (26,28,29) and was found in our study, several parameters did not show significant differences between overweight and obese children, except for a significantly higher WC in the obese group, which reinforces its importance as a risk indicator. The impaired levels of insulin, HOMA-IR, TG, HDL-C, and total- and HMW-adiponectin in the obese group compared to the normal group confirm the remarkable impact of obesity on metabolic disorders. Compared to normal and overweight children, obese children have a higher prevalence of many components of MetS. This pattern is similar to other studies in obese children and adolescents, in which low serum adiponectin levels were associated with markers of MetS, such as hyperglycemia, hyperinsulinemia, high blood pressure, and dyslipidemia (2,25,30).

When comparing our cohort, stratified by the presence or absence of MetS, there is a suggestion that the prevalence of MetS increases directly with BMI. Similar to Turkish and Portuguese children with MetS and obese Italian children, in our study, no differences were observed in TChol level, suggesting that this indicator is less critical in this age group (24,31,32). However, in Korean children, an association between non-HDL cholesterol and MetS has been reported (33).

Regarding biochemical parameters, total-adiponectin had the strongest inverse correlation respect to HMW adiponectin, with glycaemia, followed by number of MetS components, HOMA-IR and insulin concentration. Similar findings have been described, where total adiponectin had a significant inverse relation with HOMA-IR and obesity, and its low concentration was an essential determinant of insulin sensitivity and HDL in children and may predict type 2 diabetes (7,29,32,34,35). No significant correlation was found for biochemical parameters related to fat metabolism (e.g., TG, HDL-C, LDL-C and TChol), probably due to the young age of the population and the pathophysiology of MetS (24,36). Longitudinal studies showed that blood pressure and TG decreased when HOMA decreases, independently of changes in BW, supporting the hypothesis that insulin resistance is the central abnormality contributing to these cardiovascular risk factors and development of atherosclerosis and MetS (24,29). The studies found that insulin resistance, or its accomplice, hyperinsulinemia could precede to dyslipidemia, enhancing the output of very-LDL and raising TG; this lipid overload in muscle is diverted to the liver, promoting fatty liver and atherogenic dyslipidemia (37). These mechanisms affecting lipid metabolism could be at an early stage in our young population where, instead, we observed impaired glucose homeostasis as the principal affliction (38). These results obtained in the present study, support the early observations about the need to include insulin resistance, as proposed in the WHO criteria, for the diagnosis of MetS in children (23,31,39,40).

A protective role of adiponectin is evident early in life and compromised in youth-onset obesity, and low concentrations could be considered a risk factor (7,32,34). It has been suggested that low levels of adiponectin are involved in the association between childhood obesity and adult atherosclerosis (41). In the present study, total- and HMW-adiponectin were decreased in obese children and correlated with anthropometric variables (weight and BMI). However, whereas Total-adiponectin correlated with several biochemical parameters, HMW-adiponectin only correlated with HDL-C. Previous studies have found that sub-fractions of adiponectin have different biological effects, but their degree of association may vary according to the characteristics of the population, such as the different age groupings included in the studies (42,43). Adiponectin levels decline with age in association with changes in sex hormones and growth factors. Among growing youth, total fat mass is the primary determinant of adiponectin concentrations, and the age effect is mostly a result of increased fat mass with increased age (44,45). Consistent with the above, changes in total- and HMW-adiponectin levels in childhood obesity is different to that in elderly obese patients (46). Therefore, the relationship between adiponectin and the biochemical parameters of dyslipidemia may not be established until puberty (47).

Besides, the association of HMW adiponectin with MetS indicators seems to be influenced by adiposity (48). In obese prepubertal children, HMW adiponectin shows a closer relationship with the improvement of carbohydrate metabolism parameters than with body fat content. Other studies confirm that the relationships of plasma adiponectin with a favorable lipid profile depend on adiposity and that central obesity plays a significant role in the relationships of adiponectin with TG. These findings may mean that adiponectin may not necessarily play a favorable role in lipid metabolism, and it might have multiple effects on this metabolic process based on the underlying condition. Different studies have demonstrated that adiponectin concentrations have ethnic variance and were lower in Asian as compared to African-American children, were positively related to insulin sensitivity and HMW-adiponectin was not superior in predicting metabolic variables (49,50,51). Our data indicate that, in the context of the MetS in Mexican children, HMW-adiponectin might not have the same degree of relevance. Hence, the relationships between adiponectin levels and anthropometric and biochemical indicators in children appear to be independent of sex and influenced by ethnicity and lifestyles associated with modernization. We suggest that the genetic backgrounds of cohorts should also be considered in future studies and body composition analysis should be more detailed in order to investigate the relevance of adiponectin in pathogenesis of pediatric MetS.

### Study Limitations

Limitations of our study are mostly due to the limited sample size and its cross-sectional nature. However, our findings are consistent with the idea that ethnic differences influence the distribution of adiponectin isoforms and their relationship with metabolic parameters.

## Conclusion

Childhood obesity is related to several impaired biochemical parameters, including the concentration of total- and HMW-adiponectin. A low adiponectin concentration was related closely to the prevalence of MetS. The strong inverse correlation between adiponectin levels and biochemical parameters related to carbohydrate metabolism, contribute to the hypothesis that low adiponectin levels are associated with an elevated risk of diabetes. The absence of correlation between total- and HMW-adiponectin and fat metabolism indicators could be explained by the young age of the study population. Furthermore, it reinforces the importance of early insulin resistance in development of the MetS and possibly future vascular events. Therefore, circulating concentration of total adiponectin may represent an excellent biomarker to evaluate the risk of metabolic complications in young Mexican children. Additionally, a consensual pediatric definition of MetS is needed in order to better compare between studies and populations, and adequate screening and evaluation of children at risk or with MetS.

## Figures and Tables

**Table 1:**
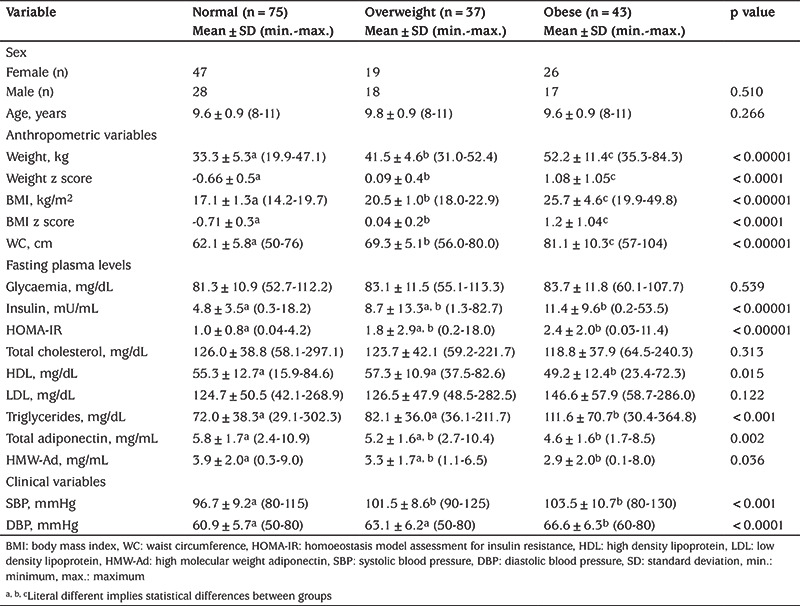
Comparison of anthropometric, biochemical, and clinical characteristics of the study participants according to obesity status

**Table 2:**
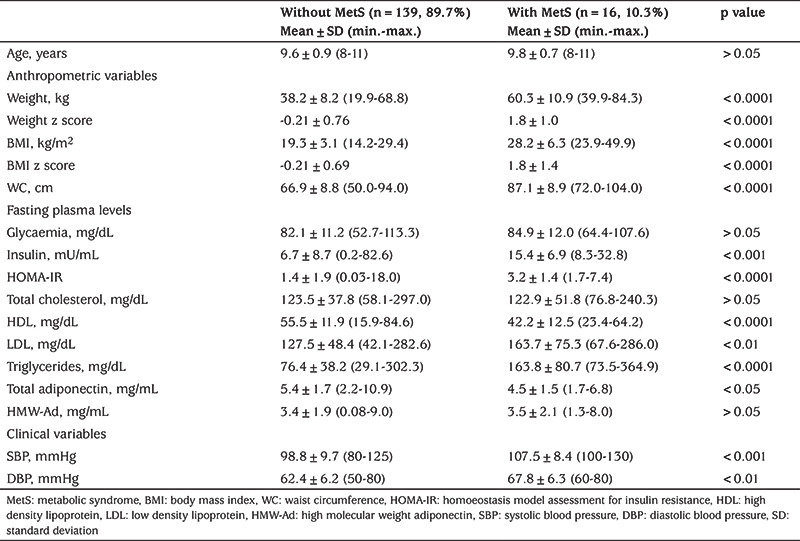
Comparison of anthropometric, biochemical, and clinical characteristics of the study participants according to the presence or absence of metabolic syndrome

**Table 3:**
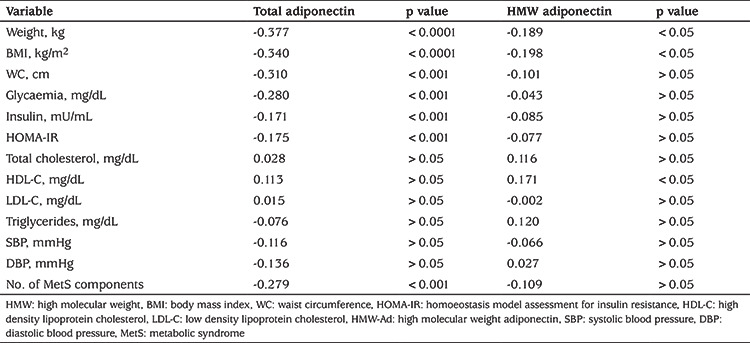
Pearson’s correlation analysis between total adiponectin, high molecular weight-adiponectin and anthropometric, clinical, and biochemical parameters in the study cohort
